# Computed Tomographic Lesion Descriptors in Foals With Hematogenous Osteomyelitis: Interobserver Agreement and Association With Outcome

**DOI:** 10.1111/vru.70231

**Published:** 2026-08-26

**Authors:** Kajsa Gustafsson, Maurizio Longo, Katrien Vanderperren, Hanna Haardt, Maarten Haspeslagh, Emanuël Stas, Donatella De Zani, Camille Buyck, Mickaël Robert, Davide D. Zani

**Affiliations:** ^1^ Department of Veterinary Medicine and Animal Sciences – DIVAS University of Milan Lodi Italy; ^2^ Diagnostic Imaging Unit CTO Veterinario Arenzano Italy; ^3^ Department of Morphology, Imaging, Orthopedics, Rehabilitation and Nutrition Faculty of Veterinary Medicine Ghent University Merelbeke Belgium; ^4^ Department of Large Animal Surgery, Anaesthesia and Orthopaedics Ghent University Merelbeke Belgium; ^5^ Equine Clinic: Surgery and Radiology Department of Veterinary Medicine Freie Universität Berlin Berlin Germany; ^6^ Centre Hospitalier Vétérinaire Équin de Livet Livarot‐Pays‐d'Auge France

**Keywords:** bone lysis, computed tomography, foal, interobserver agreement, osteomyelitis, transition zone

## Abstract

Computed tomography (CT) is increasingly used to diagnose osteomyelitis in foals, but the prognostic value and interobserver reproducibility of specific CT lesion descriptors remain unclear. This multicenter retrospective study aimed to characterize CT features of hematogenous osteomyelitis in foals using a structured radiologic feature set, assess interobserver agreement, and evaluate associations between CT descriptors and survival to hospital discharge and long‐term survival. We hypothesized that CT features considered aggressive, including a long transition zone, permeative or moth‐eaten osteolysis, and discontinuous or irregular periosteal reaction, would be negatively associated with survival. Foals diagnosed with osteomyelitis and examined by CT at three tertiary referral centers were included. CT images were independently reviewed by three blinded observers. Interobserver agreement was assessed using Krippendorff's alpha. Associations between clinical variables, CT descriptors, and survival outcomes were evaluated using logistic regression. Fifty‐six foals met inclusion criteria. Thirty‐six foals (64%) survived to hospital discharge, and 26 of 32 foals (81%) with follow‐up data survived long term. Articular involvement (*p *= 0.04; odds ratio [OR], 0.06; 95% confidence interval [CI], 0.04–0.89) and a long transition zone (*p *= 0.03; OR, 0.13; 95% CI, 0.23–0.78) were negatively associated with survival to discharge. Lesions at multiple locations were negatively associated with long‐term survival (*p *= 0.03; OR, 0.05; 95% CI, 0.04–0.77). Interobserver agreement ranged from weak to moderate. Associations between individual CT descriptors and survival were limited, highlighting the need for prospective studies and improved standardization of CT lesion descriptors in foals with osteomyelitis.

**Abbreviations**
CT: computed tomography

## 1 Introduction

Computed tomography (CT) is increasingly used for the diagnosis and preoperative planning of osteomyelitis in foals. However, the prognostic value and interobserver reproducibility of specific CT lesion descriptors have not been defined. Although the relationship between CT findings and clinical outcomes has recently been investigated, including a unicentric cohort study evaluating clinical variables, lesion location, and general CT characteristics, the association between specific CT lesion descriptors and outcome remains unexplored in the veterinary literature [1].

In small animal radiology, CT‐based characterization of osseous lesions is well established, with lesion morphology commonly used to differentiate aggressive from non‐aggressive processes. In equine practice, however, morphological assessment of bone lesions has historically relied primarily on radiography rather than CT, limiting detailed evaluation of lesion features and three‐dimensional extent. Consequently, current understanding of lesion aggressiveness, cortical involvement, and periosteal reaction patterns in equine osteomyelitis is largely extrapolated from small animal imaging or equine radiographic studies, highlighting the need for further tomographic characterization [2, 3, 4, 5].

Differentiation between aggressive and non‐aggressive bone lesions is typically based on assessment of cortical integrity, presence and pattern of bone lysis, characteristics of the periosteal reaction, and the nature of the interface between normal and abnormal bone, referred to as the transition zone [2, 3, 4, 5]. Bone lysis patterns are commonly categorized as geographic, moth‐eaten, or permeative, reflecting increasing degrees of aggressiveness. Geographic lysis is characterized by well‐defined focal bone loss, often with a sclerotic margin and short transition zone, and is generally associated with slower disease progression [6]. In contrast, moth‐eaten and permeative lysis patterns demonstrate less defined areas of bone destruction, ill‐defined transition zones, and limited osseous adaptation, consistent with more rapidly progressive lesions [2, 3, 4].

Smooth and continuous periosteal reactions are generally associated with slower biological processes and bone adaptation, including some traumatic or stress‐related conditions. In contrast, irregular or discontinuous reactions (e.g., lamellated or sunburst patterns) are more commonly observed in rapidly progressive or biologically aggressive disease [3, 6]. Similarly, a short transition zone indicates a distinct interface between normal and abnormal bone and suggests osseous adaptation, whereas a long transition zone reflects poorly demarcated margins and more aggressive biological behavior [2, 3, 5].

Although osteomyelitis in foals is relatively common and aggressive bone lesion patterns have been described, detailed CT‐based musculoskeletal descriptors, such as lysis pattern, periosteal reaction subtype, and transition zone length, and their interobserver reproducibility have not been systematically evaluated. Existing reports in foals have focused primarily on specific disease entities or general imaging findings rather than a structured, descriptor‐based approach [1, 5, 7, 8, 9, 10, 11, 12].

Parts of the study population, associated clinical variables, and general lesion characteristics have been reported previously in a unicentric cohort evaluating the clinical application and prognostic value of CT in foals with osteomyelitis [1]. In contrast to that study, the present investigation uses a structured, descriptor‐based CT assessment that includes previously evaluated lesion characteristics, such as articular and physeal involvement, articular collapse, and luxation, as well as additional descriptors, including detailed characterization of lysis pattern, transition zone, periosteal reaction subtypes, cortical destruction, pathological fracture, and soft tissue extension. In addition, interobserver agreement was formally assessed for each descriptor.

Therefore, the objectives of this study were to (1) systematically characterize CT lesion descriptors in foals with hematogenous osteomyelitis using a structured radiologic feature set, (2) quantify interobserver agreement for these descriptors, and (3) evaluate their association with survival to hospital discharge and long‐term survival.

## 2 Materials and Methods

### 2.1 Animals

Foals that were presented to Centre Hospitalier Vétérinaire Equin de Livet in France, Faculty of Veterinary Medicine University of Ghent in Belgium, and the FU of Berlin in Germany between 2019 and 2023 were included in this multicentric study if they met the following criteria: (1) age <1 year, (2) presumptive clinical diagnosis of osteomyelitis, and (3) CT examination performed as part of the diagnostic evaluation.

Foals euthanized immediately following CT examination because of financial constraints or perceived poor prognosis were included in the analysis of short‐term survival (survival to hospital discharge) but excluded from long‐term survival analyses.

Foals were excluded if clinical data did not support a diagnosis of osteomyelitis (e.g., the absence of inflammatory hematologic findings or concurrent septic disease) or if CT findings were attributed to trauma or suspected developmental orthopedic disease.

All foals were treated according to the clinical judgment and standard practices of the attending clinician at each tertiary referral center.

### 2.2 Data Collection

Clinical data were collected retrospectively using a standardized electronic data collection form. All procedures were conducted in accordance with institutional ethical guidelines and applicable data protection regulations. Data were obtained from medical records and, when necessary, through telephone follow‐up with owners to complete outcome questionnaires. Because of the retrospective study design, specific owner consent for data use was not obtained.

Admission variables included signalment (age, sex, and breed), clinical parameters (heart rate, respiratory rate, rectal temperature, mucous membrane appearance, and lameness grade), and concurrent diseases (umbilical infection, diarrhea, septic arthritis, ataxia, developmental musculoskeletal disease, and pneumonia). Clinicopathologic variables included hematologic parameters (white blood cell count) and biochemical variables (total solids concentration) [1].

Treatment data were recorded retrospectively and included treatment modality (medical management, surgical management, or euthanasia) and the rationale for treatment selection (optimal treatment, financial limitations, or poor prognosis). Surgical interventions included arthroscopic debridement, open lesion debridement, and local intralesional antimicrobial administration. Medical management consisted of systemic antimicrobial therapy, with regional limb perfusion performed when anatomically feasible. All foals undergoing surgical treatment also received systemic antimicrobial therapy [1].

Foals euthanized for financial reasons were distinguished from those euthanized for poor prognosis on the basis of whether the owner declined the recommended medical or surgical treatment [1].

CT images were independently reviewed by two board‐certified veterinary radiologists (ECVDI) and one clinician with extensive experience in large animal diagnostic imaging. Imaging data were recorded using a standardized electronic form.

### 2.3 CT Lesion Descriptors

Lesion variables included anatomical distribution (axial, appendicular, or both), specific anatomical location (coxofemoral; stifle; elbow; shoulder; tarsus; fetlock; pelvis; cervical vertebrae and lumbar vertebrae; vertebral column [cervical and lumbar vertebrae combined]; P3; carpus; ribs), and presence of single or multiple lesions [1].

A structured set of 18 CT lesion descriptors was used to evaluate the osteomyelitic lesions. All three observers independently assessed each CT examination according to these predefined variables. Representative examples of each descriptor are provided in Supporting Information Appendix 1 to support standardization and improve consistency across observers.

Lesion descriptors previously evaluated [1] included physeal involvement, articular involvement (extension to the articular margin), articular collapse, and articular luxation. CT lesion descriptors evaluated in this study included pathological fracture, cortical destruction, soft tissue extension, lysis pattern (geographic, moth‐eaten, or permeative), presence of sclerosis, transition zone length (short or long), and periosteal reaction (presence of periosteal reaction; the nature of the periosteal reaction; smooth; irregular; continuous; discontinuous) (Images 1, 2, 3 ). Observers were blinded to clinical outcomes. A descriptor was considered present when recorded by at least two of three observers. Interobserver agreement was quantified using Krippendorff's alpha, and descriptors with low agreement were interpreted cautiously in outcome analyses 4.

**FIGURE 1:**
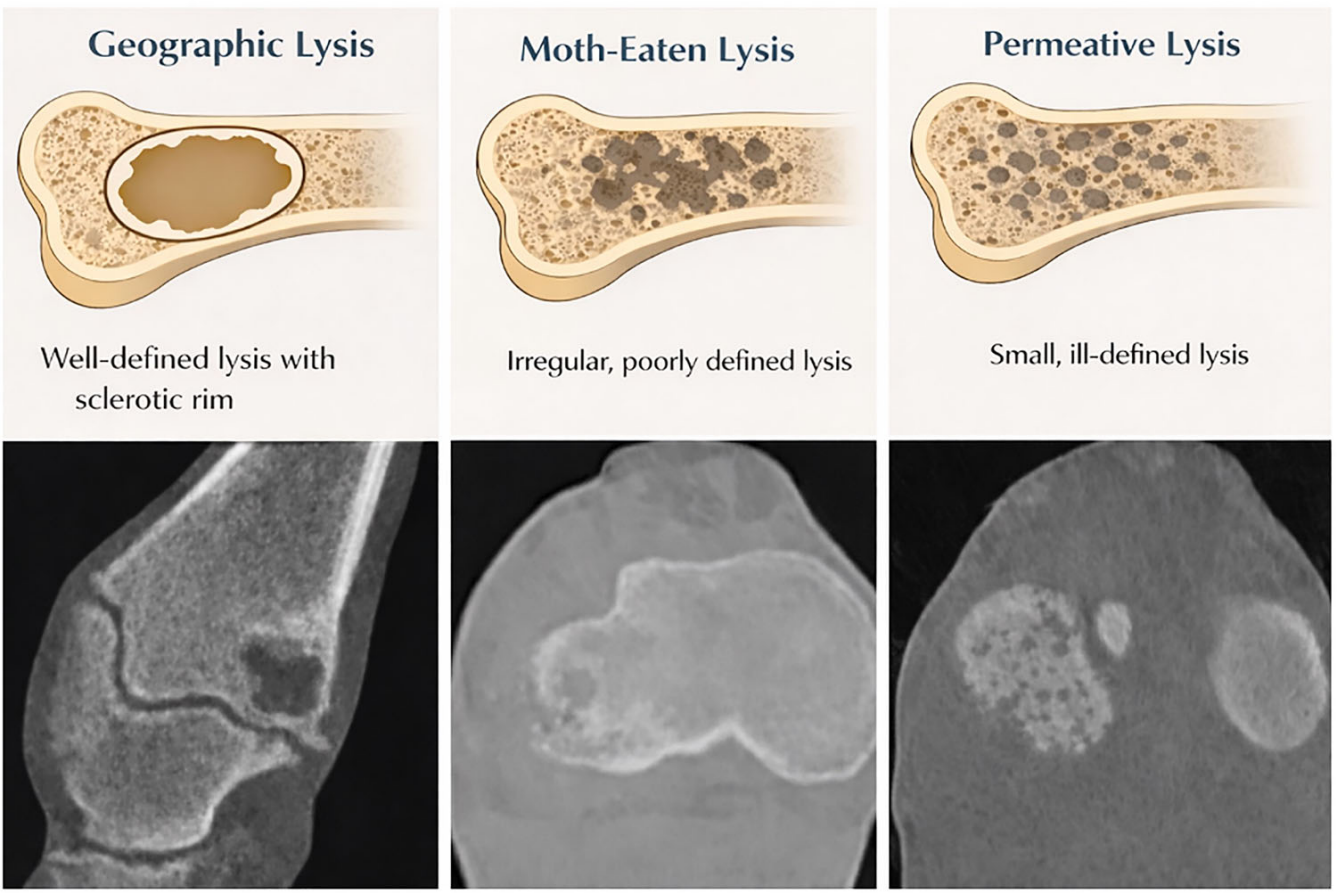
Patterns of aggressive and non‐aggressive bone lysis on schematic illustration and computed tomography. Top row: Schematic representations of three patterns of cortical and medullary bone lysis. Geographic lysis (left) is characterized by a single, well‐defined lytic lesion. Moth‐eaten lysis (center) demonstrates multiple, variably sized, poorly marginated lytic foci that coalesce. Permeative lysis (right) consists of numerous small, ill‐defined lytic defects permeating the medullary cavity. Bottom row: Corresponding computed tomographic images from representative clinical cases illustrating each lytic pattern. The left image (geographical lysis) shows a sagittal image of the distal radius, which shows a sharply marginated lytic defect with surrounding sclerosis. The center image (moth‐eaten lysis) shows a transverse image of the distal femur, which demonstrates irregular, coalescing areas of medullary bone loss with indistinct margins. The right image (permeative lysis) shows a transverse image of the distal femur, which shows multiple small, poorly defined lytic foci.

**FIGURE 2:**
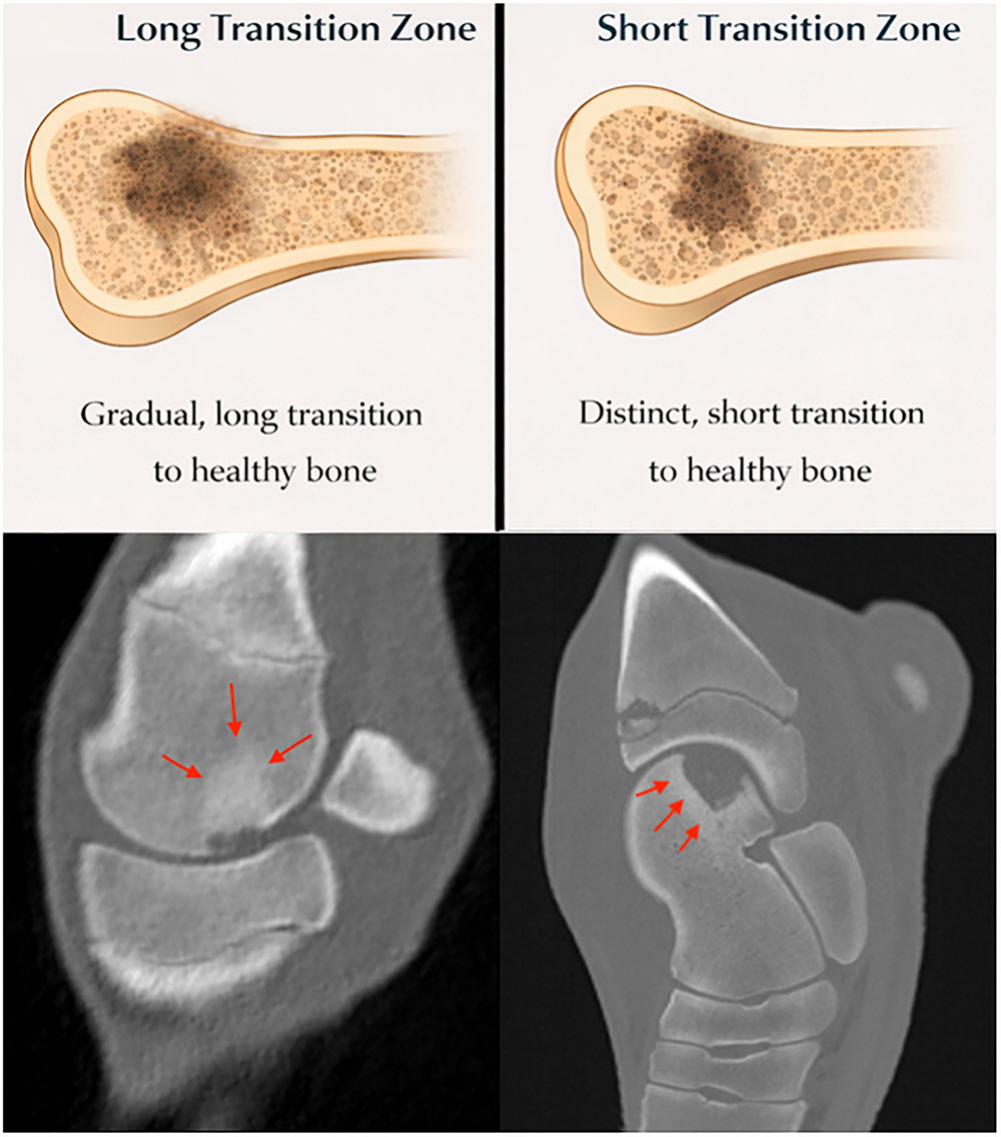
Transition zone. Top row: Schematic representations of lesions with differing zones of transition. A long zone of transition (left) is characterized by a gradual, poorly defined interface between abnormal and normal bone, reflecting a wide margin and more aggressive biological behavior. A short zone of transition (right) demonstrates a sharp, well‐defined boundary between diseased and adjacent normal bone, consistent with a narrow margin and less aggressive biological behavior. Bottom row: Corresponding sagittal computed tomographic images from representative clinical cases. The left image shows a lesion of the distal epiphysis of the distal third metacarpal bone with associated sclerosis as a long zone of transition, which shows a poorly marginated area of sclerosis with indistinct borders blending into adjacent trabecular bone (red arrows). The right image shows a lesion of the proximal aspect of the lateral trochlea of the talus with an associated short zone of transition, demonstrating a sharply demarcated subchondral lytic defect with a clearly defined interface with surrounding bone (red arrows).

**FIGURE 3:**
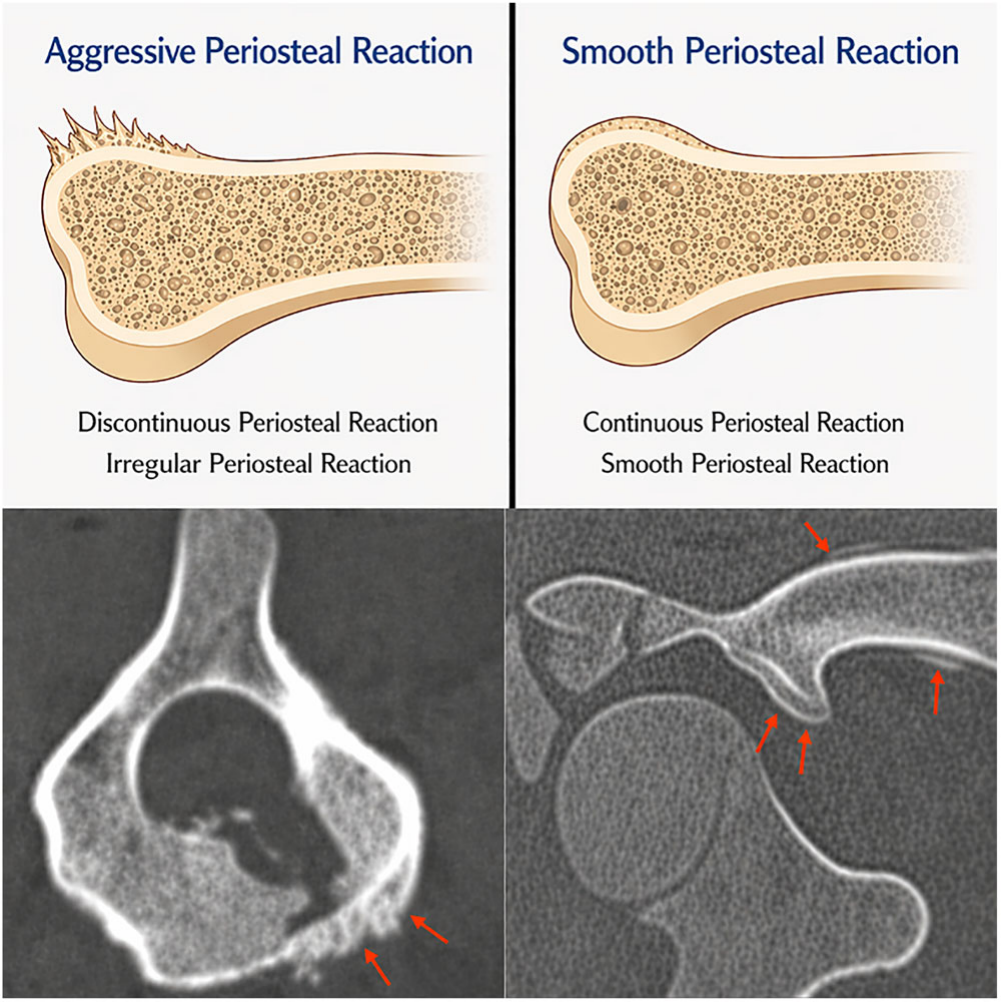
Patterns of periosteal reaction and their radiologic significance. Top row: Schematic representations of two patterns of periosteal reaction. The left image shows a periosteal reaction with both a discontinuous and irregular component. This is characterized by interrupted, spiculated new bone formation extending perpendicular to the cortex, consistent with rapid periosteal stimulation and more aggressive biological behavior. The right image shows a periosteal reaction with both a continuous and smooth component, which demonstrates uniform, uninterrupted new bone formation along the cortical surface, reflecting slower periosteal response and less aggressive disease processes. Bottom row: Corresponding transverse computed tomographic images from representative clinical cases. The left image shows a transverse image of C2. The discontinuous, irregular periosteal reaction shows interrupted and uneven periosteal new bone formation along the cortical margin (red arrows). The right image shows a transverse image of the ischium. The continuous, smooth periosteal reaction demonstrates a uniform layer of periosteal new bone paralleling the cortex (arrows).

### 2.4 Image Acquisition

CT image acquisition in Centre Hospitalier Vétérinaire Equin de Livet in France was performed using Canon Aquilion Large Bore (Canon Medical Systems, Otawara, Tochigi, Japan).

CT image acquisition in Faculty of Veterinary Medicine at the University of Ghent in Belgium was performed using 320‐slice Canon Aquilion ONE ViSION Edition (Canon Medical Systems, Otawara, Tochigi, Japan) or Lightspeed QX/I (GE Medical Systems, Milwaukee, WI, USA).

CT image acquisition in the FU of Berlin was performed in dorsal recumbency using Canon Aquilion Large Bore (Canon Medical Systems, Otawara, Tochigi, Japan).

### 2.5 Statistical Analysis

Foals were excluded only if inclusion criteria were not met. Cases with missing data were retained in the dataset and excluded only from specific analyses for which data were unavailable.

Two binary outcome variables were defined: survival to hospital discharge and long‐term survival (>1 year). Each outcome was analyzed separately. Statistical significance was set at *p* < 0.05.

Normality of continuous variables was assessed using the Shapiro–Wilk test. Univariable logistic regression was performed to evaluate associations between independent variables and each outcome. For continuous predictors, linearity of the logit was assessed. Variables with *p* < 0.20 on univariable analysis were considered for inclusion in multivariable logistic regression models.

Prior to multivariable modeling, correlations between candidate independent variables were evaluated to assess multicollinearity. A correlation coefficient ≥0.6 was considered indicative of collinearity, and only one of the correlated variables was retained in the model. Multivariable models were refined using stepwise backward elimination of nonsignificant variables, beginning with the highest *p* value, while monitoring model fit at each step.

Not all variables significant in the univariable analysis remained in the multivariable model. Variables that caused complete separation of the data were eliminated. Other variables were eliminated if they were nonsignificant and yielded the highest *p* value at their respective step. The nonsignificant variables that remained in the model remained because their elimination resulted in a relevant decrease in model fit.

Final model performance was assessed using the area under the receiver operating characteristic (ROC) curve.

Foals euthanized immediately following CT examination without treatment were excluded from long‐term survival analysis. Interobserver agreement among the three observers was assessed using Krippendorff's alpha.

All statistical analyses were performed using IBM SPSS Statistics version 29 (IBM Corp., Armonk, NY, USA).

## 3 Results

### 3.1 Study Population

Sixty clinical records were reviewed. Four cases were excluded because CT findings could not be reliably differentiated between traumatic and osteomyelitic lesions. Exclusion decisions differed among observers, and no case was excluded by all three observers. Fifty‐six foals met the inclusion criteria and were included in the final analysis. Detailed results are presented in Table 1 and Supporting Information Appendices 2–4.

**TABLE 1:** Univariable and multivariable logistic regression analyses evaluating associations between anatomical location and computed tomography (CT) lesion descriptors with survival to hospital discharge and long‐term survival.

	Survival to discharge							Long‐term survival						
		Univariate			Multivariate				Univariate			Multivariate		
	(*n*) 56	Exp. (*B*)	95% CI	*p* value	Exp. (*B*)	95% CI	*p* value	(*n*) 32 36‐4	Exp. (*B*)	95% CI	*p* value	Exp. (*B*)	95% CI	*p* value
**Anatomical data**														
Axial	13/18	1.69	0.5–5.7	0.4				7/10	0.8	0.12–5.2	0.8			
Appendicular	27/43	0.75	0.2–2.8	0.7				21/23	1.0	0.9–11	1.0			
Multiple lesions	15/28	0.39	0.15–1.2	0.1				8/11	0.6	0.006–0.6	0.015	0.53	0.004–0.8	0.03
Coxofemoral	9/12	0.39	0.45–8.0	0.4				5/8	0.6	0.9–4.1	0.6			
Stifle	5/11	0.38	0.1–1.4	0.15				3/4	0.14	0.23–0.9	0.04			
Elbow	6/8	1.8	0.3–9.9	0.5				4/5	1.4	0.13–14	0.8			
Shoulder	2/5	0.33	0.05–2.2	0.3				1/2	0.13	0.01–1.7	0.12			
Tarsus	9/16	0.61	0.19–2.0	0.4				7/8	0.7	0.13–3.7	0.7			
Fetlock	4/6	1.3	0.19–6.8	0.9				4/4	1.4	0.13–14	0.8			
Pelvis	9/12	1.9	0.5–8.0	0.4				6/8	0.8	0.12–5	0.8			
Vertebral column	6/11	0.6	0.16–2.3	0.5				3/6	0.3	0.017–5.5	0.4			
Cervical v.	3/8	0.27	0.06–1.3	0.1				0/3	*x*	*x*	*x*			
Lumbar v.	3/3	*x*	*x*	*x*				3/3	*x*	*x*	*x*			
P3	2/3	1.1	0.09–13	0.9				1/3	0.3	0.2–5.5	0.4			
Carpus	2/3	1.1	0.09–13	0.9				2/2	*x*	*x*	*x*			
Ribs	2/2	*x*	*x*	*x*				1/1	*x*	*x*	*x*			
**CT lesion descriptors**														
Moth‐eaten lysis	13/25	0.38	0.12–1.16	0.09				8/11	0.25	0.5–1.3	0.1	0.83	0.006–1.2	0.07
Permeative lysis	2/3	0.9	0.1–13	0.9				2/2	*x*	*x*	*x*			
Geographical lysis	18/24	2.3	0.7–7.4	0.15				13/18	1.9	0.4–10	0.4			
Long transition zone	9/20	0.28	0.09–0.9	0.03	0.13	0.02–0.8	0.03	6/7	0.4	0.08–2.5	0.35			
Short transition zone	27/34	5.6	1.7–18.3	0.005				20/23	*x*	*x*	*x*			
Sclerosis	33/53	*x*	*x*	*x*				24/30	*x*	*x*	*x*			
Soft tissue lesions	20/31	1.0	0.3–3.0	1.0				14/19	0.6	0.12–3	0.5			
Periosteal reaction	15/24	0.9	0.3–2.6	0.8				8/15	0.7	0.13–3.7	0.7			
Irregular periosteal reaction	11/17	1.0	0.3–3.3	1.0				5/10	0.8	0.12–5.2	0.8			
Smooth periosteal reaction	2/2	*x*	*x*	*x*				1/2	0.3	0.017–5.5	0.4			
Discontinuous periosteal reaction	12/18	1.2	0.36–3.8	0.8				6/12	1	0.16–6.4	1			
Continuous periosteal reaction	1/2	0.5	0.03–9.2	0.7				0/1	*x*	*x*	*x*			
Physeal involvement	18/32	0.43	0.14–1.4	0.15	0.5	0.1–2.2	0.35	12/17	0.3	0.05–1.7	0.17			
Articular involvement	24/43	0.1	0.13–0.88	0.04	0.06	0.1–2.2	0.04	14/24	*x*	*x*	*x*			
Articular luxation	1/4	0.16	0.02–1.7	0.12				0/1	*x*	*x*	*x*			
Articular collapse	1/7	0.07	0.007–0.6	0.02				0/1	*x*	*x*	*x*			
Pathological fracture	6/9	1.1	0.25–5.1	0.9				5/6	*x*	*x*	*x*			
Cortical destruction	33/51	1.2	0.2–8.0	0.8				23/28	1	0.9–11	1			

*Note*: Descriptive data in the survival to discharge column and the long‐term follow‐up indicate the number of foals assigned each descriptor that survived to discharge and at long‐term follow‐up. Variables with *p* < 0.05 were considered statistically significant. Variables with *p* < 0.20 on univariable analysis were included in multivariable modeling. Variables marked with “*x*” indicate insufficient data for statistical analysis.

### 3.2 Clinical Presentation

#### 3.2.1 Signalment

Of the 56 foals, 32 (57%) were male, and 24 (43%) were female. Warmbloods were the most common breed (22/56, 39%), followed by Thoroughbreds (18/56, 32%), Standardbreds (10/56, 18%), and other breeds (6/56, 11%). Median age at presentation was 39.5 days (interquartile range [IQR], 19–75 days) (Supporting Information Appendices 2 and 3).

#### 3.2.2 Clinical Parameters

Mean heart rate was 88 beats per minute (95% confidence interval [CI], 82–94), and median respiratory rate was 34 breaths per minute (IQR, 24–41). Mean rectal temperature was 38.6°C (95% CI, 38.5–38.8). Lameness, graded using the American Association of Equine Practitioners (AAEP) scale (0–5), was ≥Grade 3 in 59% of foals (Supporting Information Appendices 2 and 3).

#### 3.2.3 Clinicopathologic Findings

Median white blood cell count was 11.3 × 10^3^/µL (IQR, 9.2–16.8). Mean hematocrit was 32.6% (95% CI, 30.6–34.5), and median total solids concentration was 61 g/L (IQR, 58–64.5) (Supporting Information Appendices 2 and 3).

#### 3.2.4 Concurrent Diseases

Thirty foals (57%) had one concurrent disease at the time of CT examination, and 13 (25%) foals had more than 1 concurrent disease. Ten foals (19%) had no concurrent disease identified (Supporting Information Appendix 3).

#### 3.2.5 Diagnostic Imaging

Ten foals underwent full‐body scan, 30 foals underwent half‐body scan, and 16 foals underwent sectorial scans. A total of 100 osteomyelitic lesions were identified among the 56 foals. Twenty‐eight foals (50%) had lesions affecting multiple anatomical locations. Lesions most frequently involved the appendicular skeleton (43/56, 77%), with the tarsus representing the most common anatomical site (16/56, 29%). Associations between lesion location and survival outcomes are summarized in Table 1.

#### 3.2.6 Treatment

Medical management alone was performed in 20 foals (38%), surgical management in 16 foals (31%), and euthanasia in 16 foals (31%). In 4 foals, the treatment was unknown. Thirty‐three foals received treatment considered optimal by the attending clinician, whereas four received treatment modified because of financial constraints (Supporting Information Appendix 4).

#### 3.2.7 CT Lesion Descriptors

Associations between CT lesion descriptors and survival to hospital discharge and long‐term survival are presented in Table 1.

#### 3.2.8 Interobserver Agreement

Interobserver agreement among the three observers was assessed using Krippendorff's alpha. Agreement values for individual CT lesion descriptors are presented in Table 2.

**TABLE 2:** Interobserver agreement of each computed tomography (CT) lesion descriptor.

CT lesion descriptor	Krippendorff's alpha
Moth‐eaten lysis	0.2
Permeative lysis	−0.04
**Geographical lysis**	0.49
Long transition zone	0.23
Short transition zone	0.24
Sclerosis	0.14
Soft tissue involvement	0.03
**Periosteal reaction**	0.45
**Irregular periosteal reaction**	0.41
Smooth periosteal reaction	0.11
**Discontinuous periosteal reaction**	0.56
Continuous periosteal reaction	0.07
**Physeal involvement**	0.65
**Articular involvement**	0.54
**Articular luxation**	0.72
**Articular collapse**	0.56
Pathological fracture	0.07
**Cortical destruction**	0.47

*Note*: Descriptors with moderate or higher agreement are marked in bold. Interpretation of Krippendorff's alpha: negative—less than chance, <0.2—poor, 0.21–0.4—fair, 0.41–0.6—moderate, 0.61–0.8—good, 0.8–1.0—very good.

Overall, interobserver agreement was higher for previously described descriptors [1] than for the CT lesion descriptors introduced in this study. Among the newly evaluated descriptors, lysis pattern, sclerosis, and certain periosteal reaction subtypes demonstrated lower levels of agreement between observers.

#### 3.2.9 Prognostic Variables Associated With Outcome

Thirty‐six of 56 foals (64%) survived to hospital discharge. Four cases were lost to follow‐up, leaving 32 foals available for long‐term survival analysis. Twenty‐six of these 32 foals (81%) were alive at long‐term follow‐up (>1 year).

On univariable analysis, variables negatively associated with survival to hospital discharge included the presence of a long transition zone (*p *= 0.03), articular involvement (*p *= 0.04), and articular collapse (*p *= 0.02). A short transition zone was positively associated with survival to discharge (*p *= 0.005).

In multivariable analysis, articular involvement (*p *= 0.03) and the presence of a long transition zone (*p *= 0.04) remained significantly associated with decreased survival to discharge (Table 1; Image 4).

**FIGURE 4:**
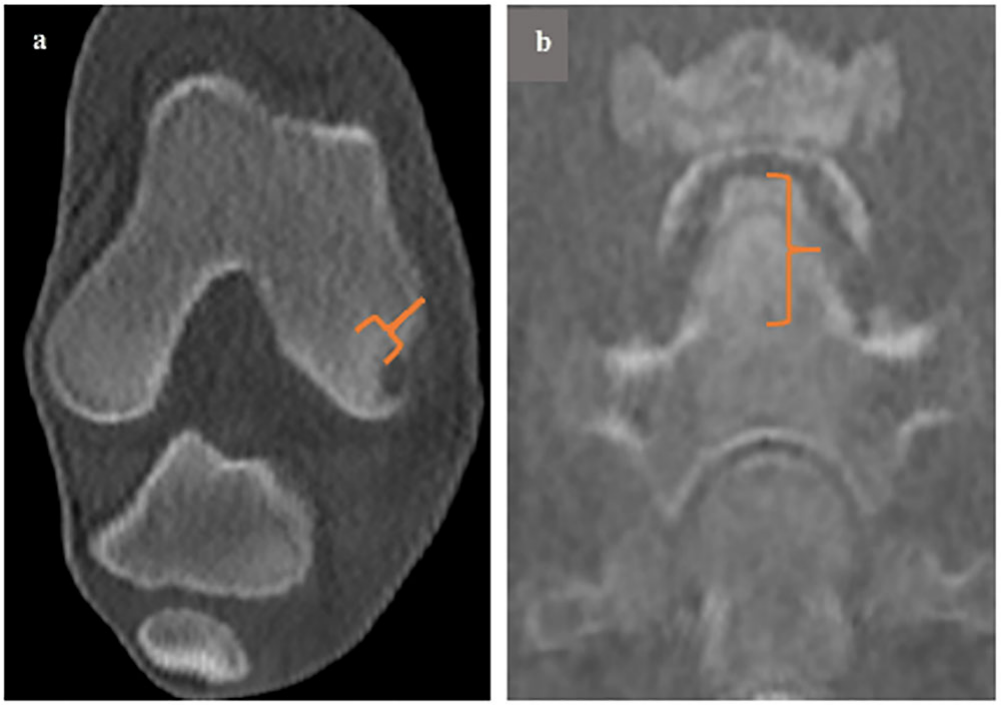
(a) Transverse image of the distal femoral condyle. Example of a **short transition zone** (orange bracket). A short and distinct transition zone is indicative of a slow or healing process, with time for the healthy bone to adapt and contain the lesion. (b) Reformatted dorsal plane image of the vertebral column focusing on C7. Example of a **long transition zone** (orange bracket). A long transition zone is indicative of a lesion that is fast‐growing with no or little adaptation of the healthy bone. This was significantly associated with decreased survival to discharge.

On univariable analysis, decreased long‐term survival was associated with a higher number of concurrent diseases (*p *= 0.009), the presence of lesions at multiple anatomical locations (*p *= 0.015), and involvement of the stifle joint (*p *= 0.04). In multivariable analysis, only the presence of lesions at multiple anatomical locations remained significantly associated with decreased long‐term survival (*p *= 0.03), Table 1.

No variable pairs yielded a correlation coefficient of 0.6 or greater, and therefore, no variable or descriptor was excluded because of multicollinearity in the multivariable analysis.

None of the foals presenting with abnormal mucous membranes, articular luxation, articular collapse, or lesions involving the cervical vertebrae survived to long‐term follow‐up. Conversely, all foals without evidence of sclerosis or articular involvement survived long term within the available follow‐up cohort, resulting in insufficient data for statistical analysis of these variables (Table 1).

Breed was not significantly associated with outcome. Survival to discharge was observed in 12/22 (55%) Warmbloods, 5/10 (50%) Standardbreds, and 15/18 (83%) Thoroughbreds. Long‐term survival was reported in 9/20 (45%) Warmbloods, 3/9 (33%) Standardbreds, and 12/17 (71%) Thoroughbreds.

## 4 Discussion

This study investigated the association between CT‐derived lesion descriptors in foals diagnosed with osteomyelitis and survival outcomes, while accounting for relevant clinical variables in these complex cases. A recent unicentric study by Buyck et al. [1] evaluated the clinical application and prognostic value of CT in foals with osteomyelitis, focusing on clinical parameters, anatomical lesion distribution, and general CT characteristics. The present work complements that study by adopting a structured, descriptor‐based CT assessment framework, with particular emphasis on detailed musculoskeletal lesion descriptors and formal evaluation of interobserver agreement.

The CT lesion descriptors applied in this study are well established in musculoskeletal radiology and are commonly used to differentiate aggressive from non‐aggressive osseous lesions [1, 2, 3, 4]. However, their systematic application to CT examinations of osteomyelitis in foals, as well as their reproducibility among observers, has not previously been reported.

Among the evaluated descriptors, the presence of a long transition zone and articular involvement were the only specific imaging variables significantly associated with decreased survival to hospital discharge. To the authors’ knowledge, this represents one of the first reports in foals with osteomyelitis in which transition zone morphology has been associated with short‐term outcomes following CT examination. Although this association was not observed for long‐term survival, it remains a potentially relevant feature when interpreting CT examinations in this population. In human medicine, a long transition zone has been associated with acute rather than chronic osteomyelitis, suggesting limited osseous adaptation to the infectious process [13]. The second descriptor significantly associated with decreased survival to discharge was articular involvement, and all foals without articular involvement survived long term. In agreement with Buyck et al. [1], articular involvement appears to be among the strongest determinants of outcome in foals with osteomyelitis undergoing CT examination.

The presence of osteomyelitis affecting multiple anatomical locations was the only variable significantly associated with decreased long‐term survival. A similar association was reported by Buyck et al. [1] in which lesion multiplicity was correlated with both decreased short‐term and long‐term survival. This association is likely multifactorial and may reflect increased disease burden, cumulative joint damage, and a higher risk of persistent or recurrent lameness.

Other specific imaging descriptors, including lysis pattern, periosteal reaction characteristics, and the presence of sclerosis, were not significantly associated with outcome. This lack of association may be partly explained by variable, and in some cases, low interobserver agreement for these descriptors. This limitation should also be considered when interpreting the association between a long transition zone and short‐term survival, as a long transition zone was associated with reduced survival to discharge in this study, but interobserver reliability for this feature was only fair (0.23–0.24). Reduced interobserver reliability is not uncommon in radiologic studies, particularly when evaluating features for which consensus definitions or validated grading systems are lacking [14, 15, 16, 17]. In one recent publication, interobserver agreement for distal interphalangeal joint pathology was similarly low (*κ *= 0.03), underscoring the subjective component inherent in veterinary imaging interpretation [15].

Because systematic application of CT descriptors has not previously been described in equine patients, this may partly explain the lower agreement observed for certain variables. Nevertheless, approximately half of the evaluated descriptors demonstrated moderate agreement [14]. Another contributing factor may be the relatively recent adoption of CT as a routine diagnostic modality in foals with osteomyelitis.

In the present study, foals received either medical or surgical management, although treatment protocols were not standardized. Medical management primarily consisted of systemic antimicrobial therapy and supportive care, whereas surgical treatment included debridement of infected bone and joints, joint lavage, and placement of local antimicrobial agents when indicated. No significant differences in survival were observed between treatment groups. This may reflect heterogeneity in lesion severity, case selection, and the relatively small sample size. It is also possible that some lesions not detected on CT resolved with antimicrobial therapy alone, highlighting the importance of thorough lesion detection, ideally including full‐body CT when feasible.

Differences between equine and small animal osteomyelitis may also influence the relevance of specific CT descriptors. In small animals, osseous lesions are less commonly intra‐articular and are frequently confined to the metaphysis due to the absence of transphyseal vessels [4]. In contrast, a substantial proportion of cases in the present study involved intra‐articular osteomyelitis, which may increase the prognostic importance of joint involvement relative to detailed descriptor‐based lesion characteristics [7, 18, 19, 20, 21, 22, 23].

Comparisons with human osteomyelitis literature further support the biological plausibility of these findings. In human medicine, CT is commonly used to assess cortical destruction, identify sequestra, and guide surgical planning, complementing MRI for early marrow assessment [24, 25]. Features consistent with aggressive bone behavior, including ill‐defined margins and long transition zones, have been associated with more acute or severe infection.

Several foals were euthanized immediately following CT examination, often prior to initiation of treatment. Although detailed documentation of owner decision‐making was limited, these outcomes were likely influenced by a combination of financial constraints, perceived prognosis, lesion severity, and welfare considerations. CT provided a comprehensive assessment of lesion extent and severity, which may have informed these decisions. However, CT findings should guide clinical decision‐making and treatment planning rather than serve as the sole justification for euthanasia.

When interpreting these findings, it should be acknowledged that treatment was not standardized and that clinician perception of prognosis may influence therapeutic decisions. Individual clinician bias and owner‐related factors, including financial considerations and perceived value of the foal, may have influenced outcomes. Differences in survival between breeds were observed descriptively, although breed was not statistically associated with outcome. Breed‐related differences in survival have been reported in other equine diseases, such as pneumonia [26], whereas other large‐scale studies have not identified breed as an independent prognostic factor [27].

Although many foals underwent full‐body CT, not all examinations included complete coverage, and some lesions may have remained undetected. Given the association between lesion multiplicity and long‐term survival, comprehensive staging may be clinically important.

As previously emphasized [28], CT should not be overinterpreted in foals. Although CT provides superior visualization of osseous lesions compared with radiography, imaging findings must be integrated with clinical evaluation, treatment feasibility, and welfare considerations when making prognostic and therapeutic decisions.

## 5 Conclusions

To the authors’ knowledge, this is one of the first multicenter studies in which structured CT lesion descriptors have been evaluated for association with survival in foals diagnosed with osteomyelitis, and in which interobserver agreement has been quantified for each descriptor.

In this cohort of 56 foals, associations between individual CT lesion descriptors and survival were generally limited, highlighting the need for continued research in this area. Significant associations with decreased survival to hospital discharge included articular involvement and the presence of a long transition zone, whereas decreased long‐term survival was significantly associated with the presence of lesions at multiple anatomical locations.

These findings support the need for further descriptor standardization and prospective evaluation to determine whether combinations of CT lesion patterns may improve prognostic stratification in foals with osteomyelitis.

## Author Contributions

**Hanna Haardt**: writing – review and editing, investigation, conceptualization. **Mickaël Robert**: conceptualization, investigation, writing – review and editing. **Katrien Vanderperren**: conceptualization, investigation, methodology, validation, writing – review and editing, supervision. **Maurizio Longo**: conceptualization, methodology, writing – review and editing, project administration, supervision, investigation, validation. **Emanuël Stas**: conceptualization, investigation, writing – review and editing. **Camille Buyck**: conceptualization, investigation, writing – review and editing. **Kajsa Gustafsson**: conceptualization, investigation, writing – original draft, methodology, validation, visualization, writing – review and editing, formal analysis, project administration, data curation, supervision. **Maarten Haspeslagh**: investigation, writing – review and editing, formal analysis, data curation. **Donatella De Zani**: writing – review and editing, conceptualization, investigation. **Davide D. Zani**: conceptualization, investigation, methodology, validation, visualization, writing – review and editing, project administration, supervision.

## Disclosure

The STROBE‐VET checklist was used for this study.

## Ethics Statement

Data were collected from the patient files and through telephone interviews with owners for completion of questionnaires. Owners did not specifically give consent to use the data due to the retrospective nature of the study. Treatment protocols were not modified due to this study.

## Conflicts of Interest

The authors declare no conflicts of interest.

## Supporting information




**Supporting File 1**: vru70231‐sup‐0001‐Appendix1.docx

**Supporting File 2**: vru70231‐sup‐0002‐Appendix2.docx

**Supporting File 3**: vru70231‐sup‐0003‐Appendix3.docx

**Supporting File 4**: vru70231‐sup‐0004‐Appendix4.docx

## Data Availability

The data that support the findings of this study are available from the corresponding author upon reasonable request.
