# Supplementary material for: Computed Tomographic Lesion Descriptors in Foals With Hematogenous Osteomyelitis: Interobserver Agreement and Association With Outcome
Source: Vet Radiol Ultrasound. 2026 Aug 26;67(5):e70231. doi: 10.1111/vru.70231 (PMC13510097; doi:10.1111/vru.70231)
Supplement: Supplementary file 1 [file VRU-67-0-s001.docx]

| **Variable** | **Median** | **Min** | **Max** | **25%** | **50%** | **75%** | **p= survival to discharge** | **Missing data** | **p= long term survival** | **Missing data** |
| --- | --- | --- | --- | --- | --- | --- | --- | --- | --- | --- |
| **Age (days)** | 39.5 | 7 | 360 | 19 | 39.5 | 75 | 0.7 | 0 | 0.9 | 4 |
| **Respiratory rate (bpm)** | 34 | 16 | 80 | 24 | 34 | 41 | **0.15** | 10 | 0.8 | 9 |
| **WBC (10*9/L)** | 11.3 | 1.1 | 44 | 9.2 | 11.3 | 16.8 | **0.175** | 11 | 0.3 | 10 |
| **Total solids g/L** | 61 | 31 | 86 | 58 | 61 | 64.5 | 0.7 | 14 | 0.9 | 11 |
| **Length of hospitalization (days)** | 4.5 | 1 | 55 | 1 | 8 | 18 | 0.9 | 3 | 0.7 | 4 |

**Appendix 2: Continuous clinical variables**

**Table 1**. Descriptive data and univariable statistical analysis of non- normally distributed continuous clinical variables and their associations with survival to discharge and long-term survival using univariable logistic regression. Variables with a p value <0.2 were included in the multivariate analysis and are marked in bold.

| **Variable** | **Mean** | **Min** | **Max** | **95%CI lower** | **95%CI upper** | **p= survival to discharge** | **Missing data** | **p= long term survival** | **Missing data** |
| --- | --- | --- | --- | --- | --- | --- | --- | --- | --- |
| **Heart rate (bpm)** | 88 | 40 | 130 | 82 | 94 | 0.4 | 9 | 0.9 | 9 |
| **Rectal temperature (°C)** | 38.6 | 37.4 | 40.3 | 38.5 | 38.8 | **0.09** | 7 | **0.18** | 7 |
| **Hematocrit (%)** | 32.6 | 18 | 46 | 30.6 | 34.5 | 0.4 | 16 | 0.34 | 13 |

**Table 2**: Descriptive data and univariable statistical analysis of normally distributed continuous physiological and hematological parameters and their associations with survival to discharge and long-term survival using univariable logistic regression. Variables with a p value <0.2 were included in the multivariate analysis and are marked in bold.
