# Supplementary material for: Computed Tomographic Lesion Descriptors in Foals With Hematogenous Osteomyelitis: Interobserver Agreement and Association With Outcome
Source: Vet Radiol Ultrasound. 2026 Aug 26;67(5):e70231. doi: 10.1111/vru.70231 (PMC13510097; doi:10.1111/vru.70231)
Supplement: Supplementary file 2 [file VRU-67-0-s002.docx]

**Appendix 1**

**CT lesion descriptors**

1. Moth-eaten lysis
2. Permeative lysis
3. Geographical lysis
4. Long transition zone
5. Short transition zone
6. Sclerosis
7. Soft tissue lesions
8. Periosteal reaction
9. Irregular periosteal reaction
10. Smooth periosteal reaction
11. Discontinuous periosteal reaction
12. Continuous periosteal reaction
13. Physeal involvement
14. Articular involvement
15. Articular luxation
16. Articular collapse
17. Pathological fracture
18. Cortical destruction

**Image 1.** Example of **motheaten lysis** (yellow arrows) involving the proximal sesamoid bones (sagittal, transverse and dorsal views). This pattern is characterized by multiple, variably sized, poorly marginated lytic foci that may coalesce, with an ill-defined transition zone and limited osseous adaptation, consistent with a more aggressive and rapidly progressive process.

**Image 2.** Example of **permeative lysis** (yellow arrows) at the level of proximal femoral metaphysis (transverse, sagittal and dorsal plane). Permeative lysis consists of numerous small, poorly defined lytic lesions permeating the medullary bone, with a wide and indistinct transition zone, indicative of highly aggressive biological behavior and rapid lesion progression

**Image 3.** Example of **geographical lysis** (orange arrows) involving the glenoid of the scapula (transverse, dorsal and sagittal views). Geographic lysis is characterized by a focal, well-defined area of bone loss, often associated with a sclerotic margin (white arrows) and a short zone of transition, reflecting a relatively slow biological process with evidence of osseous adaptation.

**Image 4.** Example of a **long transition zone** (green brackets) in a cervical vertebral body, (sagittal and dorsal planes). A long and poorly defined transition zone reflects a gradual interface between normal and abnormal bone, consistent with aggressive biological behavior and limited osseous adaptation.

Image 5. Example of a short transition zone (yellow bracket) in femoral condyle (dorsal plane). A short and well-defined transition zone represents a sharp interface between normal and abnormal bone and is typically associated with slower disease progression and effective osseous adaptation.

**Image 6.** Example of **sclerosis** (black arrowheads) surrounding an **articular** osteomyelitic lesion (white arrowheads) in the metacarpal condyle (dorsal and sagittal planes). Sclerosis is defined as increased bone attenuation reflecting reactive bone formation and may indicate chronicity or an attempt at lesion containment

-

-

**Image 7.** Example of **soft tissue involvement** in the hip region (dorsal and transverse planes). A hypoattenuating area adjacent to the osteomyelitic lesion in the ischium (light blue arrowheads) represents extension of the infectious or inflammatory process into surrounding soft tissues

**Image 8.** Example of a **periosteal reaction** (orange arrowheads) at the level of the ischium (sagittal planes). Periosteal reaction represents new bone formation along the cortical surface in response to periosteal stimulation and may vary in morphology depending on the rate of progression and the nature of the underlying disease process

**Image 9.** Example of a **smooth and continuous periosteal reaction** (yellow arrowheads) of the pelvis (dorsal and transverse planes). This pattern is characterized by uniform and uninterrupted new bone apposition along the cortex, typically associated with slower or more chronic processes that allow time for periosteal adaptation

**Image 10.** Example of an **irregular and discontinuous periosteal reaction** (yellow arrowheads) involving the second cervical vertebra (transverse and dorsal planes).This pattern is characterized by interrupted, uneven, or spiculated new bone formation and is commonly associated with rapidly progressive or biologically aggressive lesions

**Image 11.** Example of **cortical destruction** (yellow arrows) and **physeal involvement** (green arrows) at the level of the metaphyseal–epiphyseal region of the distal femur (sagittal and dorsal planes). Cortical destruction refers to the loss of cortical integrity, while physeal involvement indicates extension of the lesion into the growth plate; both features are suggestive of aggressive disease and potential structural compromise.

**Image 12.** Example of **pathological fracture** (green arrowheads)of the femoral head associated with **articular subluxation** and **articular collapse** (orange arrow) of the coxofemoral joint. These findings indicate advanced structural compromise, including mechanical failure of bone and joint instability, typically associated with severe or late-stage disease
