# Supplementary material for: Computed Tomographic Lesion Descriptors in Foals With Hematogenous Osteomyelitis: Interobserver Agreement and Association With Outcome
Source: Vet Radiol Ultrasound. 2026 Aug 26;67(5):e70231. doi: 10.1111/vru.70231 (PMC13510097; doi:10.1111/vru.70231)
Supplement: Supplementary file 3 [file VRU-67-0-s003.docx]

|  |  | (n)  56 | % of n | Survivors to discharge (n) 56 | Long term survivors (n) 36-4 | p= survival to discharge | p= long term survival |
| --- | --- | --- | --- | --- | --- | --- | --- |
| **Treatment selected** | Surgical | 16 | 31% | 13/16 | 11/13 | 0.5 | 0.8 |
|  | Medical | 20 | 38% | 18/20 | 13/18 |  |  |
|  | Euthanasia | 16 | 31% | x | x |  |  |
| Missing |  |  |  | 4 | 5 |  |  |
| **Treatment selection basis** | Optimal treatment | 33 | 66% | 28/33 | 21/24 | 0.45 | 0.8 |
|  | Financial restraints | 4 | 8% | 2/4 | 2/2 |  |  |
|  | Euthanasia based on poor prognosis | 13 | 26% | x | x |  |  |
| Missing |  | 6 |  |  | 10 |  |  |

**Appendix 4.** Univariable statistical analysis of categorical data; chosen treatment types and their associations with short- and long-term survival using univariable logistic regression. Variables with a p value <0.2 were included in the multivariate analysis and are marked in bold. Variables marked with x did not obtain enough data to perform statistical analysis.
