# Supplementary material for: Computed Tomographic Lesion Descriptors in Foals With Hematogenous Osteomyelitis: Interobserver Agreement and Association With Outcome
Source: Vet Radiol Ultrasound. 2026 Aug 26;67(5):e70231. doi: 10.1111/vru.70231 (PMC13510097; doi:10.1111/vru.70231)
Supplement: Supplementary file 4 [file VRU-67-0-s004.docx]

| **Variable** |  | N out of 56 | % | **p= survival to discharge** | **Missing data** | **p= long term survival** | **Missing data** |
| --- | --- | --- | --- | --- | --- | --- | --- |
| **Sex** | Male | 32 | 57% | 0.9 | 0 | 0.8 | 4 |
|  | Female | 24 | 43% |  |  |  |  |
| **Breed** | Thoroughbred | 18 | 32% | **0.16** | 0 | 0.6 | 4 |
|  | Standardbred | 10 | 18% |  |  |  |  |
|  | Warmblood | 22 | 39% |  |  |  |  |
|  | Others | 6 | 11% |  |  |  |  |
| **Lameness score** | 0 | 7 | 15% | **0.16** | 13 | 0.6 | 12 |
|  | 1 | 5 | 11% |  |  |  |  |
|  | 2 | 4 | 8% |  |  |  |  |
|  | 3 | 10 | 22% |  |  |  |  |
|  | 4 | 13 | 28% |  |  |  |  |
|  | 5 | 4 | 9% |  |  |  |  |
| **Mucous membranes** | Normal | 48 | 98% | 0.7 | 7 | x |  |
|  | Abnormal | 1 | 2% |  |  | (no survivors) |  |
| **Concurrent diseases** | 0 | 10 | 19% | **0.17** | **3** | **0.009** | **3** |
|  | 1 | 30 | 57% |  |  |  |  |
|  | 2 | 6 | 11% |  |  |  |  |
|  | 3 | 6 | 11% |  |  |  |  |
|  | 4 | 0 | 0% |  |  |  |  |
|  | 5 | 1 | 2% |  |  |  |  |

**Appendix 3**. Univariable statistical analysis of categorical and ordinal data; clinical characteristics, concurrent diseases and their associations with survival to discharge and long-term survival using univariable logistic regression. Variables with a p value <0.2 were included in the multivariate analysis and are marked in bold. Variables marked with an x did not obtain enough data for analysis.
